# Evaluation of Prediction Models of the Microwire EDM Process of Inconel 718 Using ANN and RSM Methods

**DOI:** 10.3390/ma15238317

**Published:** 2022-11-23

**Authors:** Dorota Oniszczuk-Świercz, Rafał Świercz, Štefan Michna

**Affiliations:** 1 Institute of Manufacturing Technology, Warsaw University of Technology, 02-524 Warsaw, Poland; 2 Faculty of Mechanical Engineering, Jan Evangelista Purkyně University in Ústí nad Labem, 400 01 Usti nad Labem, Czech Republic

**Keywords:** wire electrical discharge machining, surface topography, RSM, ANN, Inconel

## Abstract

Precise machining of micro parts from difficult-to-cut materials requires using advanced technology such as wire electrical discharge machining (WEDM). In order to enhance the productivity of micro WEDM, the key role is understanding the influence of process parameters on the surface topography and the material’s removal rate (*MRR*). Furthermore, effective models which allow us to predict the influence of the parameters of micro-WEDM on the qualitative effects of the process are required. This paper influences the discharge energy, time interval, and wire speed on the surface topography’s properties, namely *Sa*, *Sk*, *Spk*, *Svk*, and *MRR*, after micro-WEDM of Inconel 718 were described. Developed RSM and ANN model of the micro-WEDM process, showing that the discharge energy had the main influence (over 70%) on the surface topography’s parameters. However, for *MRR*, the time interval was also significant. Furthermore, a reduction in wire speed can lead to a decrease in the cost process and have a positive influence on the environment and sustainability of the process. Evaluation of developed prediction models of micro-WEDM of Inconel 718 indicates that ANN had a lower value for the relative error compared with the RSM models and did not exceed 4%.

## 1. Introduction

The application of new micro-mechanisms to materials that are difficult to cut, such as nickel superalloys, requires the development of new machining technologies that will allow us to obtain the appropriate dimensional accuracy in terms of the microelements and achieve the desired surface quality. One of the technologies that allow the production of geometrically complex microelements made of nickel superalloys is wire electrical discharge machining. In WEDM, electric discharges occur between the wire electrode and the workpiece, causing the material to be locally removed by melting and evaporation as a result of the impact of the thermal processes of the discharge [1,2,3,4]. In order to meet the demands of shape and dimensions accuracy of micro parts, a branch of WEDM, micro-WEDM, is used. The main advance of micro WEDM is the possibility to obtain a smaller gap and corner radius in manufacturing micro parts by using the micro discharge energy (<10 mJ) per pulse and wire with a diameter below/equal to 100 μm. However, using thin wires in the micro-WEDM process is required to conduct new research that will allow for effective control of the process.

Applications of microwire electrical discharge machining in precision manufacturing of microparts in materials that are difficult to cut, such as Inconel 718, are limited by the properties of the surface texture and the low material removal rate. Currently, research is being conducted on understanding the influence of the parameters and conditions of machining on the qualitative effects of WEDM [5,6,7,8]. Nevertheless, two major groups of parameters of WEDM have the most important influence on the process. The first group of parameters is the factors that have an influence on the discharge energy. Research conducted by Chen et al. [9] indicated that an increased open voltage leads to an increase in the discharge energy and ultimately causes an increase in the amount of material removed during discharge. Ishfaq et al. [10] indicated that the discharge current has a strong influence on the cutting speed and kerf width. The second important factor that has a strong influence on the parameters of WEDM is the conditions of machining. Ilkhchi et al. [11] indicated that in addition to the parameters that define the discharge energy, the dielectric flow has an influence on the process of material removal. The turbulent dielectric flow in the gap increases the efficiency of flushing in the gap and removing molten material from the craters, which have a strong influence on the surface morphology. Research carried out by Vijayakumar et al. [12] indicated that the material of the wire electrode has a strong influence on the morphology and roughness of the surface. Using a zinc-coated brass electrode leads to a decrease in surface roughness compared with uncoated brass. Furthermore, research carried out by Abhilash et al. [13] indicated that using zinc-coated brass allowed an increase in the cutting speed. Among the treatment conditions of the micro-WEDM process that significantly affect the morphology and roughness of the surface is the number of trim offsets. Sharma et al. [14] and Burek et al. [15] indicated that surface morphology significantly depends on the number of machining passes. The use of additional passes allows a decrease in the parameters of surface roughness. Research carried out by Ali et al. [16] indicated that by using dry micro-WEDM, it was possible to reduce the size of the kerf during cutting, which had a strong influence on the geometry of the cut micro parts.

Because of the complexity of the physical phenomena occurring during electrical discharge machining, a significant part of the research has focused on the development of predictive models for the process. One of the most frequently used methodologies that allow us to determine the relationship between the input factors and the results of process optimization are the response surface methodology [17,18,19,20,21], artificial neural networks [22,23,24], desirability functions [25,26,27,28], the fuzzy possibility approach [29,30], and gray relational analysis [31,32,33]. The study provided by Jatakar et al. [34] shows that using the ANN algorithm can effectively diagnose and self-monitor complex manufacturing processes without human intervention. Furthermore, the study conducted by Patli et al. [35] provides that the Deep learning algorithm based on the ANN models used to monitor the manufacturing process had a higher accuracy than models developed with Machine Learning (ML) classifiers. Therefore, using the ANN algorithm for building prediction model processes is the main part of Industry 4.0.

The surface topography after machining has a strong influence on fatigue strength and other tribological properties [36,37,38]. Depending on the type of treatment used, the properties of the surface topography may differ significantly despite similar values of the typical parameters describing surface roughness, such as Ra. From a tribological point of view, it is necessary to apply additional parameters to characterize the surface obtained [39,40,41,42]. In the production of micro parts, the properties of the surface topography will have even more influence on the fatigue strength. Research conducted so far on WEDM of the Inconel 718 alloy [43,44,45] has shown that many factors have an influence on the surface roughness and the material removal rate. Relatively few studies [46,47,48] have described the influence of the parameters of micro-WEDM on the surface topography’s properties. However, the influence of the discharge energy, the time interval, and the wire speed on the surface topography and the material removal rate after micro-WEDM of Inconel 718 has not been considered enough. Furthermore, industrial applications of microwire EDM require prediction models, which would allow us to choose favorable parameters to archive specific features of the topography and the *MRR*. For that reason, the main goal of this study was to determine the impact of the discharge energy, the time interval, and wire speed on the surface topography and the material removal rate. In order to achieve this goal, prediction models of micro-WEDM of Inconel 718 with RSM and ANN were developed. The main purpose of applying two different methodologies for building the predictive models was to evaluate the influence of the methodology on the accuracy.

## 2. Materials and Methods

### 2.1. Workpiece Material

Heat-resistant superalloys based on nickel are increasingly being used in the aviation, space, chemical, and energy industries. Inconel alloys are widely used for the production of turbine blades, discs, shafts, and valves. Because of its properties, such as its low thermal conductivity (11.4 W/mK), good mechanical properties, and a high tendency to work hardening, Inconel 718 has been classified as a material that is difficult to cut. The chemical composition of Inconel 718 is presented in Table 1.

### 2.2. Experimental Setup

This study focused on analyzing the influence of the parameters of micro-WEDM on the surface topography’s properties and the material removal rate. The research was divided into several stages. In the first stage of the research, the ranges of stable machining parameters were determined with no wire breaks during cutting. The second stage was an analysis of the influence of the discharge energy, the time interval, and wire speed on the properties of the surface topography of Inconel 718 after machining. In the next stage, prediction models for microwire EDM using the response surface methodology (RSM) and an artificial neural network (ANN) were developed. In the final stage, the prediction models were evaluated.

In order to achieve these tasks, experimental studies were carried out on the Charmilles Robofill 440 WEDM machine (GF Solutions, Bienne, Switzerland). The samples of the Inconel 718 alloy for cutting had dimensions of 10 × 10 × 5 mm, the electrode was a brass wire with a diameter of 0.1 mm, and deionized water was used as the dielectric.

**Figure 1:**
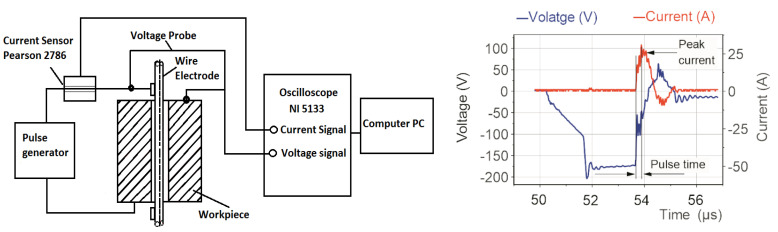
Schematic illustration of the measuring circuit with the registered current and voltage waveforms.

In the first stage of research, a measurement circuit consisting of a NI5133 oscilloscope card (National Instruments, Austin, TX, USA), a current sensor (Pearson, London, UK), and a voltage probe (Tektronix, UK Ltd., Berkshire, UK) was developed for analyzing the discharge current and the voltage waveforms (Figure 1).

The material removal process in Micro WEDM has a complex nature and depends on many parameters and conditions. The classification of parameters considers the division into parameters influencing the discharge energy, parameters related to the working electrode such as wire speed and wire tension, and processing conditions characterizing the method and pressure of the dielectric supply. Preliminary research has shown that for the machine control system adopted here, some of the processing parameters are not separate from each other. A change in the pulse time causes a simultaneous change in the discharge current’s value. An analysis of the registered voltage and the current waveforms allowed us to establish a relationship between the pulse time and the discharge current and to calculate the discharge energy according to Equation (1):

(1) $$E=\int_{0}^{t_{on}}U\left(t\right)\cdot I\left(t\right)dt\;\;\;\;\left(\mathrm{mJ}\right),$$

where:

*U*—average discharge voltage,
*I*—the height of the peak current during discharging,
*t_on_*—pulse time.

In micro-wire electrical discharge machining, the material is removed from the workpiece due to a series of discharges occurring in the gap. Material is removed from the workpiece during the pulse time ton. Next, during the time interval toff, the eroded material is removed from the gap between the working electrode and the workpiece. The time interval is responsible for the stabilization of the condition in the gap between discharges. Furthermore, have an influence on the frequency of discharges.

Parameters related to the working electrode, such as wire speed, influence the shape of the discharge carter and surface topography [49]. During the WEDM process, the wire electrode is rewound from the spool to prevent wear of the electrode, which influences the stability of the process. Furthermore, reducing wire speed can decrease the cost process and positively impact the environment and sustainability of the process.

By considering the above relations, the following parameters were chosen in the experimental studies: discharge energy, time interval, and wire speed. Table 2 presents the conditions of the experiment.

Experimental studies on the influence of the discharge energy *E*, the time interval *t_off_*, and the wire speed *WS* on the parameters describing properties of the surface topography and the material removal rate of Inconel 718 after micro-WEDM were conducted using Hartley’s experimental design with three five-level parameters. Table 3 shows the levels of the machining parameters used in the experiment. Using five-level DOE allows for investigation in a wide range (five levels) influence input parameters on the investigated output. For example, in the preliminary research, the range of parameters for micro-WEDM of Inconel 718 was established for criteria: stable discharges (observed current and voltage waveforms) without the wire breaking.

The parameters of the surface texture were measured with the Talysurf series 2 scanning profilometer (Taylor Hobson, Leicester, UK). For each cutting surface, an area of 1 × 3 mm was measured in a discretization step (10 μm), as well as the Y-axis and X-axis. The EDS spectrum of the cutting surface was investigated on a JEOL JCM-7000 NeoScope (Tokyo, Japan).

The material removal rate was calculated by dividing the lateral surface area of the cut sample by the cutting time:

(2) $$MRR=\frac{l\times h}{\Delta t}\;\left[\frac{{\mathrm{mm}}^{2}}{\min}\right]$$

where *l* is cutting length, *h* is sample height, and  Δt is the time of manufacturing.

**Figure 2:**
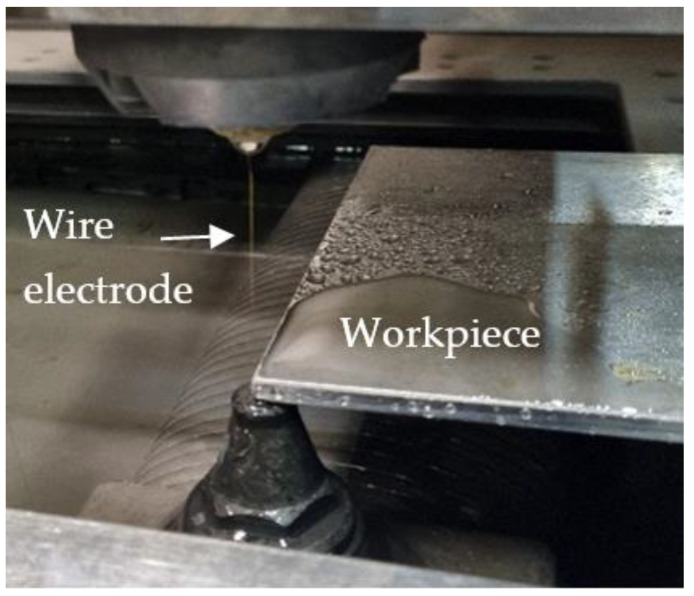
Experimental setup.

**Figure 3:**
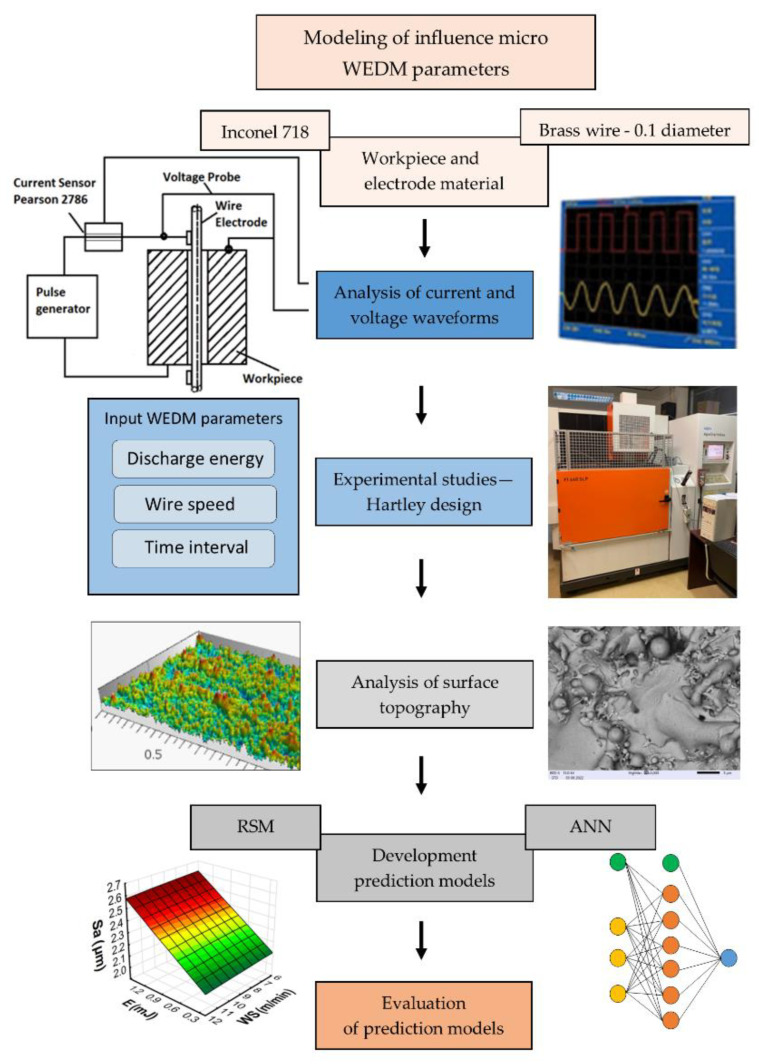
The overall framework of experiments.

The data obtained from experimental studies of the micro-WEDM of Inconel 718 were used to build predictive models of the process using the response surface methodology and artificial neural networks. Next, an evaluation of the newly developed predictive models was conducted by comparing the predictive errors. The scheme of the experimental setup is presented in Figure 2, and the overall framework of experiments is presented in Figure 3.

## 3. Results and Discussion

### 3.1. Analysis of the Surface Topography

**Figure 4:**
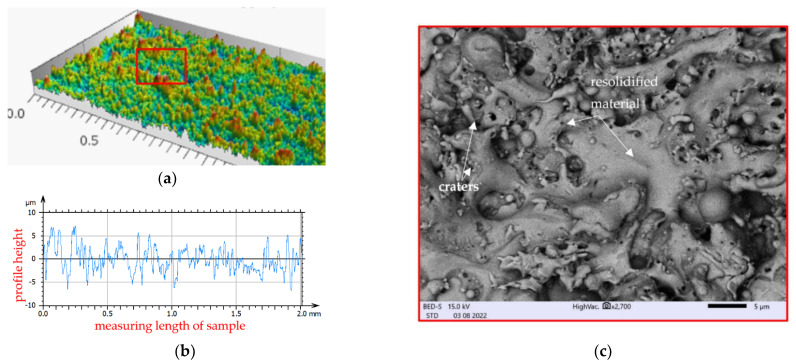
Surface topography of Inconel 718 after micro-WEDM with the parameters *E* = 1.46 mJ, *WS* = 9 m/min, and *t_off_* = 8 µs: (**a**) 3D texture; (**b**) 2D profile of measured surface; (**c**) SEM image.

In the micro-WEDM process, the material is removed as a result of the impact of the electric discharges occurring in the gap between the working electrode (in the form of a thin wire) and the surface of the workpiece. As a result of the heat flux from the discharges, a small amount of material melts and evaporates on the surface of the electrodes. At the end of the discharges, the plasma channel collapses, and some part of the melted material which was not evaporated resolidifies on the surface. In the place of the discharge, a crater occurs. Overlapping craters create a specific topography of the surface (Figure 4).

**Figure 5:**
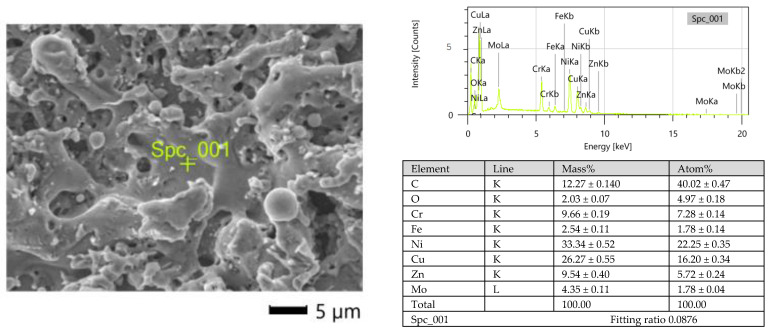
EDS spectrum of the surface of Inconel 718 after micro-WEDM: *E* = 1.46 mJ, *WS* = 9 m/min, and *t_off_* = 8 µs.

Electrical discharge occurs in the gap, leading to the melting of both the working electrode and the workpiece. The intense boiling and evaporation of the electrode material during discharge causes the diffusion of this material to the resolidified layers. An EDS analysis of the surface topography after the micro-WEDM process indicated the presence of elements from the working electrode on the processing surface (Figure 5).

**Figure 6:**
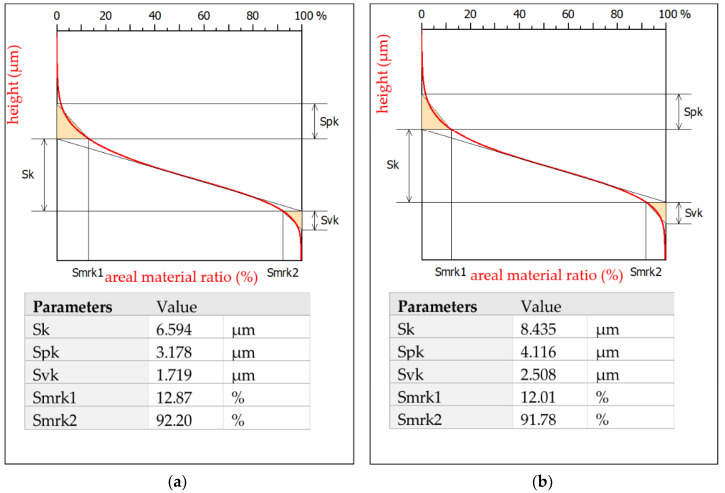
Abbott-Firestone curves after micro-WEDM of Inconel 718: (**a**) *E* = 0.21 mJ, *WS* = 9 m/min, and *t_off_* = 8 µs; (**b**) *E* = 1.46 mJ, *WS* = 9 m/min, and *t_off_* = 8 µs.

The surface topography after micro-WEDM has a complex structure. In order to describe the surface’s properties, the following functional parameters were chosen: the arithmetic means of the deviations from the mean *Sa* (the average value of the absolute height over the entire surface), *Sk* (the roughness of the core), *Spk* (the roughness of the peak), and *Svk* (the roughness of the valleys). The roughness parameters *Sk*, *Spk*, and *Svk* describe the load capacity of the surface (Figure 6). The roughness parameter *Svk* and that of the lower bearing surface (Smrk2) give information about the surface’s lubrication properties, i.e., the ability of fluid to flow through the sliding surfaces. The roughness of the peak (*Spk*) can give information about the surface’s resistance to abrasion. The higher the *Spk* value is, the lower the resistance to abrasion. The roughness of the core (*Sk*) determines the depth of the roughness after the initial breaking-in period.

The roughness of the peaks and valleys after micro-WEDM of Inconel 718 was *Spk* = 3.178 μm and *Svk* = 1.719 μm, and *Spk* = 4.12 μm and *Svk* = 2.508 μm for machining at minimal (*E* = 0.21 mJ) and maximal discharge energy (*E* = 1.46 mJ), respectively. Changing the discharge energy significantly affected the properties of the surface topography. For the sample manufactured with the lowest discharge energy (*E* = 0.21 mJ), the roughness of the peaks and core was *Spk* = 3.2 μm and *Svk* = 1.7 μm, respectively (Figure 6b). The parameter values obtained here were almost 30% lower than those of manufacturing with the maximal value of discharge energy (*E* = 1.46 mJ).

Experimental studies of the influence of the parameters of micro-WEDM on the surface topography’s properties and the *MRR* were performed according to the design of the experimental methodology of Hartley experiments with five levels and three parameters. Eighteen samples were manufactured and measured (including four replications at the center point of the plan). The average results of the experimental studies are presented in Table 4.

The surface roughness *Sa* was in the range of 2.10–2.67 µm. The roughness of the peak (*Spk*) was in the range of 3.11–4.12 µm. The roughness of the core (*Sk*) was in the range of 6.64–8.43 µm, and the roughness of the valleys (*Svk*) was in the range of 1.71–2.51 µm. The material removal rate (*MRR*) was in the range of 27.18–110.94 mm^2^/min.

### 3.2. Predictive Models

Development of the predictive models of the micro-WEDM process was carried out using the response surface methodology (RSM) and artificial neural networks (ANN). The main purpose of applying two different methodologies was to evaluate the influence of the methodology on the accuracy of the prediction model.

In the study, the investigated input parameters of micro-WEDM were the discharge energy E, the time interval (*t_off_*), and the wire speed (*WS*); the output parameters were the surface roughness (*Sa*), the roughness of the core (*Sk*), the roughness of the peak (*Spk*), the roughness of the valleys (*Svk*), and the *MRR*.

#### 3.2.1. Response Surface Methodology

The response surface methodology allows the building of predictive models based on regression and ANOVA analyses. The response surface was calculated according to the following equation:

*Y* = *f* (*E*,*t_off_*,*WS*) ± *ε*, (3)

where *Y* is the investigated response (*Sa*, *Spk*, *Svk*, or *MRR*; *f* is the regression function; *E* (discharge energy), *t_off_* (time interval), and *WS* (wire speed) are independent parameters; and *ε* is the experimental error.

In the first step of building the prediction models, different regression functions were used. On the basis of an analysis of the values of the determination coefficient *R*^2^ and the Fisher test, it was found that the best match to the results of the experimental research was obtained for the second-degree polynomial function. In the next step, analysis of variance (ANOVA) was used to develop the final regression equation. At the 95% coefficient level, the significance of each factor in the regression model was checked. If the calculated probability value Prob > f for the single factor was higher than 0.05, this meant that the factor was nonsignificant and was removed from the final regression function. The ANOVA results for surface roughness (*Sa*), the roughness of the core (*Sk*), the roughness of the peak (*Spk*), the roughness of the valleys (*Svk*), and the *MRR* are presented in Table 5, Table 6, Table 7, Table 8 and Table 9, respectively and are included in the Supplementary Materials.

Table 5 shows the ANOVA results for surface roughness (*Sa*). The calculated contribution indicated that the discharge energy had the greatest influence on the surface roughness (74.14%), followed by the time interval (14.08%) and the square of the time interval (6.78%). The ANOVA results presented in Table 6 indicate that the greatest influence on the roughness of the peak (*Spk*) was the discharge energy (70.96%). The variable with the second-greatest effect was the wire speed (*WS*) (21.51%), followed by the time interval (7.53%). Table 7 presents the ANOVA results for the roughness of the core (*Sk*). The results indicate that discharge energy (78.15%), followed by the time interval (8.32%), had the most influence on *Sk*. The contributions of the other variables to the roughness of the core (*Sk*) were significant but less important. The ANOVA results for the roughness of the valleys (Table 8) indicate that the parameter with the greatest influence on *Svk* (76.15%) was the discharge energy, followed by the square of the time interval (9.14%) and the time interval (8.28%). The ANOVA results for the material removal rate (Table 9) indicated that the time interval had the greatest influence on *MRR* (46.97%), followed by the discharge energy (36.44%).

The Fisher coefficients calculated for each regression model presented in Table 5, Table 6, Table 7, Table 8 and Table 9 was significantly greater than 1, which proves that the model was statistically significant for the selected 95% confidence level. The value of determination coefficient *R*^2^ and the adjusted coefficient of determination (*R-Adj*) for the *Sa*, *Svk*, and the *MRR* models were over 92% and 90%, respectively, and those for *Spk* and *Sk* were over 84% and 81%, respectively. The response function developed here had a very good fit with the experimental results.

The final version of the developed response function for the surface roughness (*Sa*), the roughness of the core (*Sk*), the roughness of the peak (*Spk*), the roughness of the valleys (*Svk*), and the *MRR* are described by the following polynomial functions:

*Sa* = 3.307 + 0.4068 *E* − 0.0003 *WS*^2^ − 0.2832 *t_off_* + 0.0155 *t_off_*^2^ (μm) (4)

*Spk* = 2.83 + 0.774 *E* − 0.002 *WS*^2^ − 0.0020 *WS t_off_* (μm) (5)

*Sk* = 4.90 + 2.4876 *E* − 0.6531 *E*^2^ +0.7470 *WS* − 0.0450 *WS*^2^ − 0.33859 *t_off_* + 0.0183 *t_off_*^2^ (μm) (6)

*Svk* = 3.83 − 0.0547 *E* − 0.0787 *WS* − 0.3698 *t_off_ +* 0.0208 *t_off_*^2^ 0.06954 *E WS* (μm) (7)

*MRR* = 203.59 + 88.214 *E* + 17.5524 *E*^2^ − 0.0232 *WS*^2^ − 40.3947 *t_off_ +* 2.2594 *t_off_*^2^ − 8.0758 *E t_off_* (mm^2^/min) (8)

**Figure 7:**
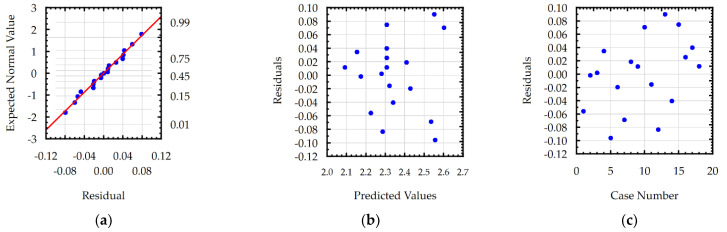
Plots for the model of *Sa*: (**a**) normal plot of the residuals, (**b**) the residuals versus the predicted values, and (**c**) the residuals versus the case number.

In the last step of the analysis of developed models, residual analyses were performed. For example, the results of the residual analyses are presented in Figure 7 for the *Sa* roughness. The normal plot of the residuals (Figure 7a) shows that the residuals are distributed along a straight line, which proves that the distribution is normal. The analyses in Figure 7b,c indicate that the residuals have a stochastic nature and that the errors are independent of each other and the case number. Analyses of the residuals performed for each model confirmed the statistical adequacy of the models.

#### 3.2.2. Artificial Neural Network

**Figure 8:**
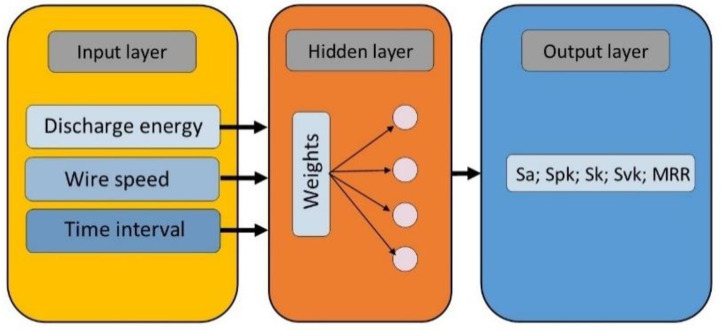
Schematic of the ANN’s architecture.

In the second part of building the prediction models for the investigated process, the ANN was used. Currently, there are many different types of ANN [50,51,52,53]. One type of ANN that allows for the complex nonlinear nature of the process is the multilayer perceptron network (MLP). After an analysis of the process in order to determine the relationship between the input data and a single output parameter, we decided to use the network architecture with three layers: the input layer with three neurons, the hidden layer with different numbers of neurons, and the output layer with one neuron (Figure 8).

The type of activation function and the number of neurons in the hidden layer were chosen experimentally. In order to avoid overfitting the ANN, several points were taken into account. The allocation of the results into the training, testing, and verification sets was achieved stochastically. The testing and verification data were equal to 20% of each of the databases and were not used for training. To prevent bias and properly build the prediction model, the holdout validation approach was provided [54]. The hold-out data, 20% the database, was only used in the final assessment of the model. The number of iterations during the learning process was set to 100 by the Broyden-Fletcher-Goldfarb-Shanno (BFGS) algorithm [55].

**Figure 9:**
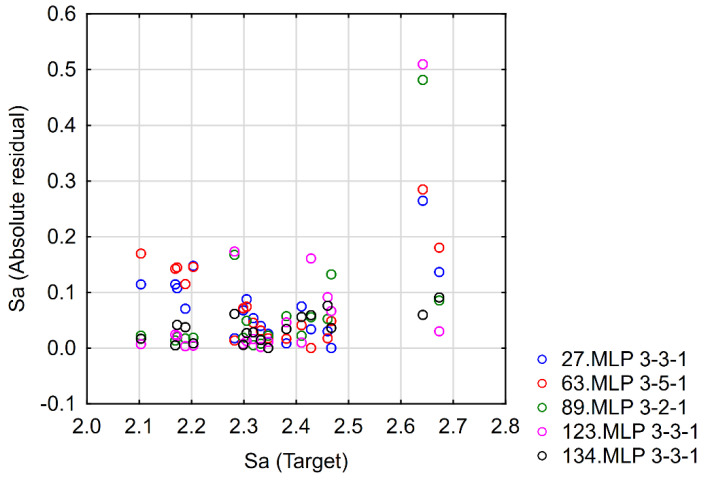
Residual analysis for different MLP architectures for a model of *Sa*.

Selection of the predictive model was performed after an analysis of the architecture of the model and the values of calculated error for the training, testing, and validation data. Models with the smallest number of neurons in the hidden layer were chosen with nearly the same value of the fitting error to test the data. Next, an analysis of the residuals was performed to choose the final architecture of the ANN. If the residuals for the test data were much higher than those for the training data, this meant that the prediction ability of the model was poor (good results were found for only the training data), which indicated an overfitting problem. An example of the residual analysis for the *Sa* model with different numbers of neurons in the hidden layer is presented in Figure 9.

The final architecture for the ANN predictive model for surface roughness (*Sa*), the roughness of the core (*Sk*), the roughness of the peak (*Spk*), the roughness of the valleys (*Svk*), and the *MRR* are presented in Table 10.

The architecture of ANN models developed for *Sa*, *Spk*, *Sk*, *Svk*, and the *MRR* did not have the ability to directly evaluate which input factor had the main influence on the investigated parameters. It was, therefore, important to carry out a sensitivity analysis for the input variables, which provided information about the contribution of the independent variables (discharge energy (*E*), wire-speed (*WS*), and time interval (*t_off_*)) to the prediction model. The sensitivity analysis was the result of the quotient of the error calculated for the investigated ANN model network without one variable and the error calculated for the model with all variables. The sensitivity analysis results presented in Table 11 indicate that the factor with the greatest influence on the parameters describing the surface topography (*Sa*, *Spk*, *Sk*, and *Svk*) was the discharge energy, followed by the wire speed and the time interval. However, in the case of the material removal rate, the factor with the greatest influence was the time interval, then the discharge energy, followed by the wire speed.

### 3.3. Evalutaion of the Predcitve Models

One of the main goals of this research was to evaluate the accuracy of the method, which allowed us to build prediction models which described the influence of the discharge energy, wire speed, and time interval on the parameters describing the topography of the surface and the material removal rate. In this study, two different methods were used: the response surface methodology and an artificial neural network. Each of the investigated methods can be evaluated on the basis of the statistical correlation coefficient, e.g., Pearson’s coefficient R (Table 12), which gives information about the fit of the models to the results of the experimental research.

The values of the correlation coefficient R obtained for both the ANN and RSM models were above 0.9, which indicated that developed models are very good at representing the variability of the investigated output factors.

The results indicated that in almost every case, the value of Pearson’s coefficient R was higher for the ANN than for the RSM. Therefore, the developed ANN models should be characterized by a lower prediction error. However, Pearson’s coefficient R does not allow for a more precise comparison of the accuracy of the models. For this reason, in addition to these analyses, the relative error of the prediction models was calculated according to the following equation:

(9) $$Error=\frac{Absolut\;residual}{Experimental\;value\;}\times 100\;\left(\%\right)$$

**Figure 10:**
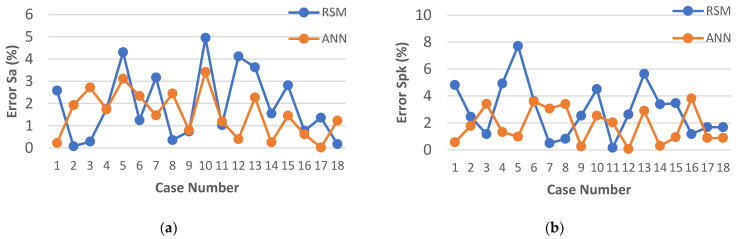
Plots of the models’ prediction error: (**a**) *Sa*; (**b**) *Spk*.

**Figure 11:**
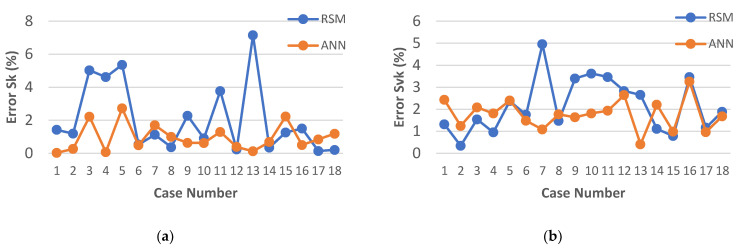
Plots of the models’ prediction error: (**a**) *Sk*; (**b**) *Svk*.

**Figure 12:**
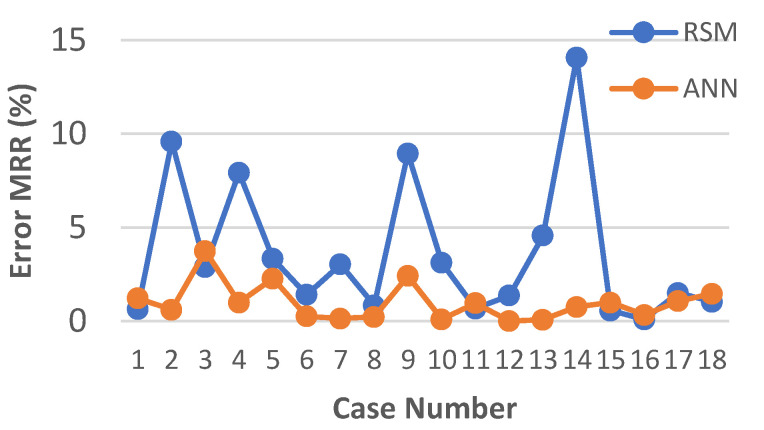
Plots of the models’ prediction error for *MRR*.

The results of calculating the relative error for each of the models developed by RSM and ANN methodology are presented in Figure 10, Figure 11 and Figure 12. An analysis of the data showed that for each model created through the use of artificial neural networks, a lower value of the relative error was obtained.

In most cases, for the models developed with ANN, the relative error of the fit did not exceed 3%, which can be considered a very good result. The largest single relative error was obtained for the parameters *Sk* and *Svk* (Figure 10b and Figure 11b). However, the error did not exceed 4%.

In the case of the models developed with the use of RSM, the lowest relative error of fit was obtained for the parameters *Sa* and *Sk* and did not exceed 5% (Figure 10a and Figure 11b). For the parameters *Svk* and *Spk*, the maximum relative error did not exceed 8% (Figure 10b and Figure 11a). Nevertheless, the highest relative value of the error for the fit of the results calculated by the model to the experimental results was observed for the *MRR* and amounted to 14% (Figure 12).

**Figure 13:**
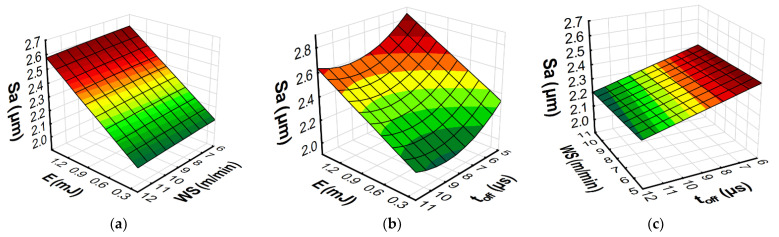
Estimated response plots for the RSM model of roughness (*Sa*): (**a**) constant *t_off_* = 8 µs, (**b**) constant *WS* = 9 m/min, and (**c**) constant *E* = 0.7 mJ.

**Figure 14:**
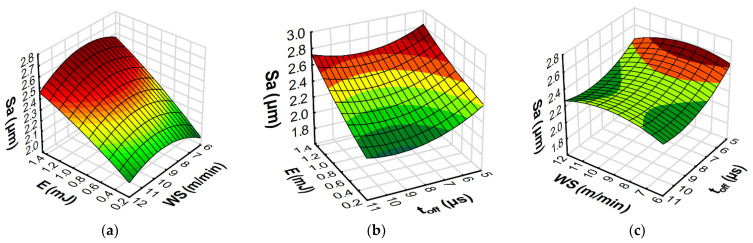
Estimated response plots for the ANN model of roughness (*Sa*): (**a**) constant *t_off_* = 8 µs, (**b**) constant *WS* = 9 m/min, and (**c**) constant *E* = 0.7 mJ.

**Figure 15:**
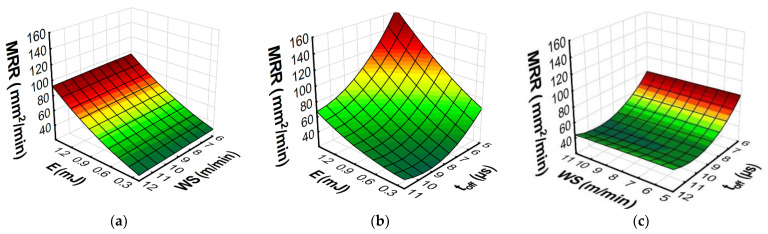
Estimated response plots for the RSM model of *MRR*: (**a**) constant *t_off_* = 8 µs, (**b**) constant *WS* = 9 m/min, and (**c**) constant *E* = 0.7 mJ.

**Figure 16:**
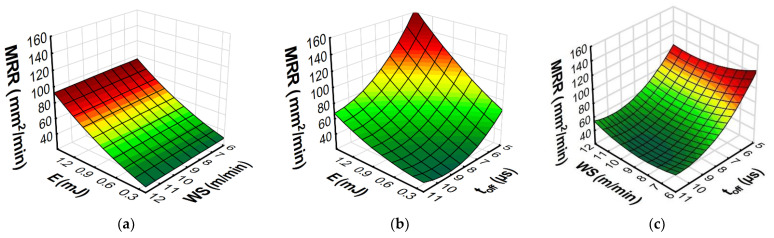
Estimated response plots for the ANN model of *MRR*: (**a**) constant *t_off_* = 8 µs, (**b**) constant *WS* = 9 m/min, and (**c**) constant *E* = 0.7 mJ.

The developed models with RSM and ANN methods allowed us to predict the influence of the input variables of microwire electrical discharge machine of Inconel 718 on the parameters of surface topography (*Sa*, *Spk*, *Sk*, and *Svk*) and the *MRR*. Furthermore, the response plots (Figure 13, Figure 14, Figure 15 and Figure 16) show the relationships between the parameters of WEDM and the output factors.

The surface roughness (*Sa*) is a parameter that determines the average height of the surface roughness and indirectly describes the average height of the craters formed on the surface of the material by an electric discharge. The dependencies presented in Figure 14 and Figure 15 indicate that the energy of electric discharge had the greatest impact on the value of the *Sa* parameter. The increase in the discharge energy for the WEDM apparatus used in the tests depended on the increase in both the current’s intensity and the impulse time. As a result, in accordance with the Gaussian heat flux in the plasma channel [1], this caused an increase in the volume of material removed in a single pulse. The dependencies presented in Figure 13b and Figure 14b indicate that the wire speed did not significantly affect the surface roughness (*Sa*). From the point of view of the sustainable development of micro-WEDM of Inconel 718, it is important to reduce the wear of the working electrode by limiting the wire speed. The time interval (Figure 13c and Figure 14c) is responsible for stabilizing the conditions in the gap. For short time intervals, arc discharge can occur, which leads to an increase in surface roughness (*Sa*). The dependencies of the effect of the discharge energy, the wire speed, and the time interval also apply to the other parameters describing the height of the roughness. The amount of material removed in a single discharge of energy determines the height of the roughness of the core (*Sk*), the roughness of the peak (*Spk*), and the depth of the roughness of the valleys (*Svk*). The plots of the estimated response functions of *Spk*, *Sk*, and *Svk* for RSM and ANN are included in the Supplementary Materials.

An analysis of the RSM and ANN prediction models of the material removal rate and its graphical interpretation (Figure 15 and Figure 16) indicated that the discharge energy and the time interval made a large contribution to the *MRR* during the micro-WEDM of Inconel 718. *MRR* increased with an increase in the discharge energy and a decrease in the time interval (Figure 15b and Figure 16b). With an increase in discharge energy, the amount of material removed in a single discharge rose, leading to an increase in the *MRR*. These results coincide with the research of Esteves et al. [56] describing the volume of material removed by a single discharge. The time interval had a direct influence on the frequency of the discharges. An increase in the time interval led to a decrease in the frequency of discharges and ultimately decreased the *MRR*.

This research conducted into micro-WEDM of Inconel 718 has indicated that the wire speed had the least influence on the *MRR* (Figure 15a,c and Figure 16a,c). Determining the optimal wire speed is important to avoid the effects of wear on the working electrode, which can lead to vibration of the wire and wire breakage.

The results indicate that for the investigated range of wire speeds, the use of speeds of 5 m/min allowed high cutting efficiency to be achieved. A decrease in the wire speed leads to decreased electrode consumption, which has a significant impact on the environment and sustainability.

## 4. Conclusions

In the present work, our main attention was on the analytical and experimental investigation of the influence of the discharge energy, wire speed, and time interval on the parameters describing the properties of the surface topography (*Sa*, *Spk*, *Sv*, *Svk*, and *MRR*) during micro-WEDM of Inconel 718. Furthermore, prediction models of the micro-WEDM process were developed via the response surface methodology and an artificial neutral network. In the final stage of research, an evaluation of the accuracy of the developed prediction was carried out. The following conclusions were drawn.

1. Discharge energy made the main contribution to the surface roughness (*Sa*, *Spk*, *Sv*, and *Svk*) and the *MRR* during micro-WEDM of Inconel 718.
2. The time interval made the main contribution to the *MRR*, as the decrease in the time interval increased the frequency of the discharge. Furthermore, for the adopted range, the time interval had the least influence on the parameters describing the surface topography’s properties.
3. Wire-speed had the least influence on the parameters describing the surface topography’s properties (*Sa*, *Spk*, *Sv*, and *Svk*) and *MRR*. Furthermore, for the lowest wire speed, it was possible to obtain a high *MRR* and a low value of surface roughness. A decrease in the wire speed led to a decrease in the consumption of the electrode, which would have a significant impact on the environment and sustainability.
4. The predictive models based on RSM and ANN for the micro-WEDM of Inconel 718 can be applied to construct technological tables for the investigated process.
5. The models developed with ANN had a lower value for the relative error compared with the RSM models. The maximum relative error for the ANN models did not exceed 4%.

## Figures and Tables

**Table 1:** Chemical composition of Inconel 718 (mass %).

Ni	Cr	Nb	Mo	Ti	Al	Co	C	Mn	Si	P	S	B	Fe
50–55	17–21	4.75–5.5	2.8–3.3	0.65–1.15	0.2–0.8	<1.0	<0.015	<0.5	<0.35	<0.015	<0.015	<0.06	Balance

**Table 2:** Machining conditions.

Electrode	Brass wire, diameter 0.1 mm
Workpiece material	Inconel 718
Height of specimen	5 mm
Discharge energy	0.21–1.46 mJ
Time interval *t_off_*	5–11 μs
Open voltage *U*_0_	220 V
Dielectric	Deionized water
Wire mechanical tension	0.2 daN

**Table 3:** The design of the experimental matrix.

Level	Parameter		
	Discharge Energy *E* (mJ)	Wire Speed (m/min)	Time Interval *t_off_* (µm)
−1.68	0.21	6	5
−1	0.42	7	6
0	0.70	9	8
1	1.04	11	10
1.68	1.46	12	11

**Table 4:** Design of the experimental matrix, with the results of the experimental studies.

Exp. No.	WEDM Input			Observed Values				
	Discharge Energy *E* (mJ)	Wire Speed *WS* (m/min)	Time Interval *t_off_* (μs)	*Sa* (μm)	*Spk* (μm)	*Sk* (μm)	*Svk* (μm)	*MRR* (mm^2^/min)
1.	0.42	7	7	2.169	3.11	6.987	1.984	45.74
2.	0.42	7	10	2.172	2.967	6.792	1.923	34.4
3.	0.426	11	6	2.282	3.085	6.645	1.94	55.12
4.	0.42	11	10	2.188	3.183	6.806	1.796	32.56
5.	1.05	7	6	2.46	3.619	7.717	2.296	100.7
6.	1.05	7	10	2.41	3.271	7.793	2.181	58.18
7.	1.05	11	6	2.467	3.599	7.852	2.257	97.02
8.	1.05	11	10	2.428	3.804	7.616	2.271	56.72
9.	0.21	9	8	2.103	3.178	6.594	1.719	27.88
10.	1.46	9	8	2.673	4.116	8.435	2.508	91.62
11.	0.74	6	8	2.305	3.223	7.589	2.129	58.64
12.	0.74	12	8	2.203	3.28	6.967	2.012	55.42
13.	0.74	9	5	2.642	3.853	8.401	2.364	101.94
14.	0.74	9	11	2.299	3.511	7.277	2.058	31.2
15.	0.74	9	8	2.381	3.64	7.648	2.021	44.1
16.	0.74	9	8	2.332	3.473	7.498	1.938	43.81
17.	0.74	9	8	2.346	3.574	7.541	1.982	43.2
18.	0.74	9	8	2.318	3.574	7.567	1.968	44.31

**Table 5:** ANOVA table for *Sa* (after elimination).

Source	Sum of Squares	Degrees of Freedom	Mean Square	*F*-Value	Prob > *f*	Contribution %
Model	0.3506	4	0.0876	39.96		
*E*	0.2599	1	0.2599	355.59	0.0003	74.14
*WS* ^2^	0.0174	1	0.0174	23.93	0.0163	4.99
*t_off_*	0.0493	1	0.0493	67.54	0.0037	14.08
*t_off_* ^2^	0.0237	1	0.0237	32.52	0.0106	6.78
Error	0.0021	13				
Total SS	0.3527	17	*R-sqr* = 0.92		*R-Adj* = 0.90	

**Table 6:** ANOVA table for *Spk* (after elimination).

Source	Sum of Squares	Degrees of Freedom	Mean Square	*F*-Value	Prob > *f*	Contribution %
Model	1.2631	3	0.4210	16.31		
*E*	0.8963	1	0.8963	48.73	<0.0001	70.96
*WS* ^2^	0.2717	1	0.2717	14.77	0.0017	21.51
*t_off_*	0.0950	1	0.0950	5.16	0.0392	7.53
Error	0.0142	14				
Total SS	1.5206	17	*R-sqr* = 0.84		*R-Adj* = 0.81	

**Table 7:** ANOVA table for the *Sk* (after elimination).

Source	Sum of Squares	Degrees of Freedom	Mean Square	*F*-Value	Prob > *f*	Contribution %
Model	4.1954	6	0.6992	58.51		
*E*	3.2785	1	3.2785	823.14	<0.0001	78.15
*E* ^2^	0.0988	1	0.0988	24.80	0.0155	2.36
*WS*	0.1761	1	0.1761	44.21	0.0069	4.20
*WS* ^2^	0.2511	1	0.2511	63.04	0.0041	5.99
*t_off_*	0.3491	1	0.3491	87.65	0.0025	8.32
*t_off_* ^2^	0.0416	1	0.0416	10.44	0.0481	0.99
Error	0.0119	11				
Total SS	4.2073	17	*R-sqr* = 0.87		*R-Adj* = 0.85	

**Table 8:** ANOVA table for the *Svk* (after elimination).

Source	Sum of Squares	Degrees of Freedom	Mean Square	*F*-Value	Prob > *f*	Contribution %
Model	0.6621	5	0.1317	36.96		
*E*	0.5196	1	0.5196	437.61	0.0002	78.92
*WS*	0.0120	1	0.0120	10.15	0.0498	1.83
*t_off_*	0.0545	1	0.0545	45.90	0.0065	8.28
*t_off_* ^2^	0.0601	1	0.0601	50.66	0.0057	9.14
*E WS*	0.0120	1	0.0120	10.16	0.0497	1.83
Error	0.0035	12				
Total SS	0.6585	17	*R-sqr* = 0.95		*R-Adj* = 0.92	

**Table 9:** ANOVA table for *MRR* (after elimination).

Source	Sum of Squares	Degrees of Freedom	Mean Square	*F*-Value	Prob > *f*	Contribution %
Model	9016.25	6	1502.70	10.52		
*E*	3285.90	1	3285.90	253.14	<0.0001	36.44
*E* ^2^	175.54	1	175.54	13.52	0.0036	1.95
*WS* ^2^	318.09	1	318.09	24.50	0.0004	3.53
*t_off_*	4234.76	1	4234.76	326.24	<0.0001	46.97
*t_off_* ^2^	867.89	1	867.89	66.86	<0.0001	9.63
*E WS*	134.07	1	134.07	10.32	0.0082	1.49
Error	142.78	11				
Total SS	9159.03	17	*R-sqr* = 0.98		*R-Adj* = 0.98	

**Table 10:** The architecture of the ANN for the predictive models.

Model	Number of Neurons in the Hidden Layer	Activation Function in the Hidden Layer	Activation Function in the Output Layer	Optimization Algorithm
*Sa*	3	Exponential	Logistic	BFGS 0
*Spk*	2	Tanh	Tanh	BFGS 45
*Sk*	4	Identity	Tanh	BFGS 3
*Svk*	2	Exponential	Exponential	BFGS 10
*MRR*	3	Logistic	Tanh	BFGS 50

**Table 11:** Sensitivity analysis of the input variables.

Model	Values of Sensitivity		
	*E*	*WS*	*t_off_*
*Sa*	3.05	1.28	1.16
*Spk*	6.35	5.43	1.91
*Sk*	7.19	1.57	1.75
*Svk*	5.14	1.08	1.40
*MRR*	335.87	22.68	360.77

**Table 12:** Sensitivity analysis for the input variables.

Model	Correlation Coefficient R			
	*RSM*		*ANN*	
		Train	Test	Validation
*Sa*	0.96	0.97	0.98	0.99
*Spk*	0.92	0.96	0.99	0.99
*Sk*	0.93	0.95	0.97	0.98
*Svk*	0.97	0.97	0.98	0.99
*MRR*	0.99	0.99	0.99	0.99

## Data Availability

The data presented in this study are available on request from the corresponding authors. The data are not publicly available due to privacy.

## Supplementary Materials

The following supporting information can be downloaded at: https://www.mdpi.com/article/10.3390/ma15238317/s1, Figure S1: Estimated response plots for RSM model of roughness *Sa*; Figure S2: Estimated response plots for ANN model of roughness *Sa*; Figure S3: Estimated response plots for RSM model of roughness *Spk*; Figure S4: Estimated response plots for ANN model of roughness *Spk*; Figure S5: Estimated response plots for RSM model of roughness *Sk*; Figure S6: Estimated response plots for ANN model of roughness *Sk*; Figure S7: Estimated response plots for RSM model of roughness *Svk*; Figure S8: Estimated response plots for ANN model of roughness *Svk*; Figure S9: Estimated response plots for RSM model of MRR; Figure S10: Estimated response plots for ANN model of MRR.
