# Supplementary material for: Evaluation of Prediction Models of the Microwire EDM Process of Inconel 718 Using ANN and RSM Methods
Source: Materials (Basel). 2022 Nov 23;15(23):8317. doi: 10.3390/ma15238317 (PMC9737947; doi:10.3390/ma15238317)
Supplement: Supplementary file 1 [file materials-15-08317-s001.zip › materials-2003749-supplementary.pdf]

Evaluation of Prediction Models of the Microwire EDM Process of Inconel 718 Using ANN and RSM Methods

Dorota Oniszczuk-Świercz [1,*], Rafał Świercz [1] and Štefan Michna [2]

[1] Institute of Manufacturing Technology, Warsaw University of Technology, 02-524 Warsaw, Poland
[2] Faculty of Mechanical Engineering, Jan Evangelista Purkyně University in Ústí nad Labem, 400 01 Usti nad Labem, Czech Republic
* Correspondence: dorota.swiercz@pw.edu.pl

The developed models with RSM and ANN methods allowed us to predict the influence of the input variables of microwire electrical discharge machining of Inconel 718 on the parameters of surface topography (*Sa, Spk, Sk, and Svk*) and the *MRR*. Furthermore, the response plots (Figures S1–S10) show the relationships between the parameters of WEDM and the output factors.

The surface roughness (*Sa*) is a parameter that determines the average height of the surface roughness and indirectly describes the average height of the craters formed on the surface of the material by an electric discharge. The dependencies presented in Figures S1 and 2 indicate that the energy of electric discharge had the greatest impact on the value of the *Sa* parameter. The increase in the discharge energy for the WEDM apparatus used in the tests depended on the increase in both the current’s intensity and the impulse time. As a result, in accordance with the Gaussian heat flux in the plasma channel [1], this caused an increase in the volume of material removed in a single pulse. The dependencies presented in Figures S1b and S2b indicate that the wire speed did not significantly affect the surface roughness (*Sa*). From the point of view of the sustainable development of micro-WEDM of Inconel 718, it is important to reduce the wear of the working electrode by limiting the wire speed. The time interval (Figures S1c and S2c) is responsible for stabilizing the conditions in the gap. For short time intervals, arc discharge can occur, which leads to an increase in surface roughness (*Sa*). The dependencies of the effect of the discharge energy, the wire speed, and the time interval also apply to the other parameters describing the height of the roughness. The amount of material removed in a single discharge of energy determines the height of the roughness of the peak (*Spk*) (Figures S3 and S4), the roughness of the core (*Sk*) (Figures S5 and S6), and the depth of the roughness of the valleys (*Svk*) (Figures S7 and S8).

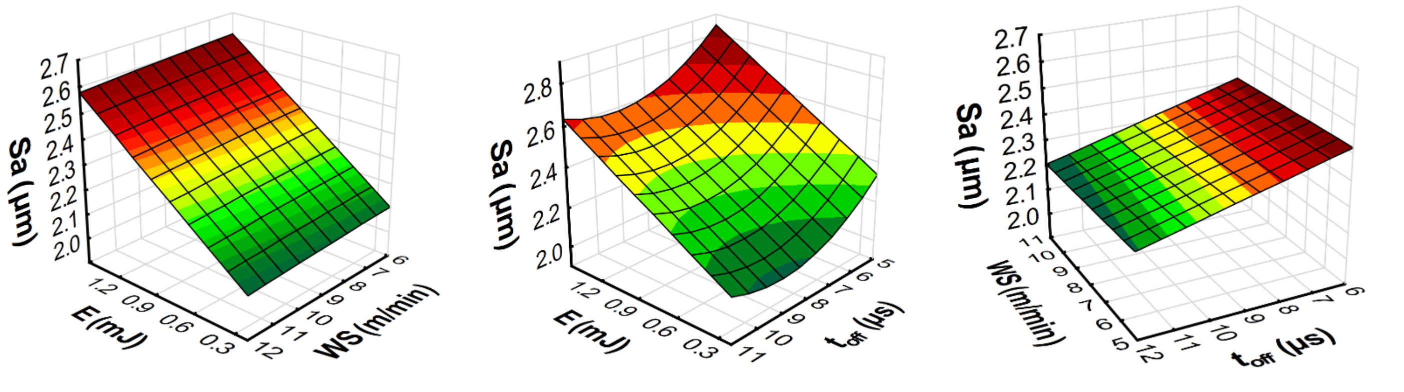

(a) (b) (c)

Figure S1. Estimated response plots for RSM model of roughness *Sa*: (a) constant t_{off} = 8 µs; (b) constant WS = 9 m/min; and (c) constant E = 0.7 mJ

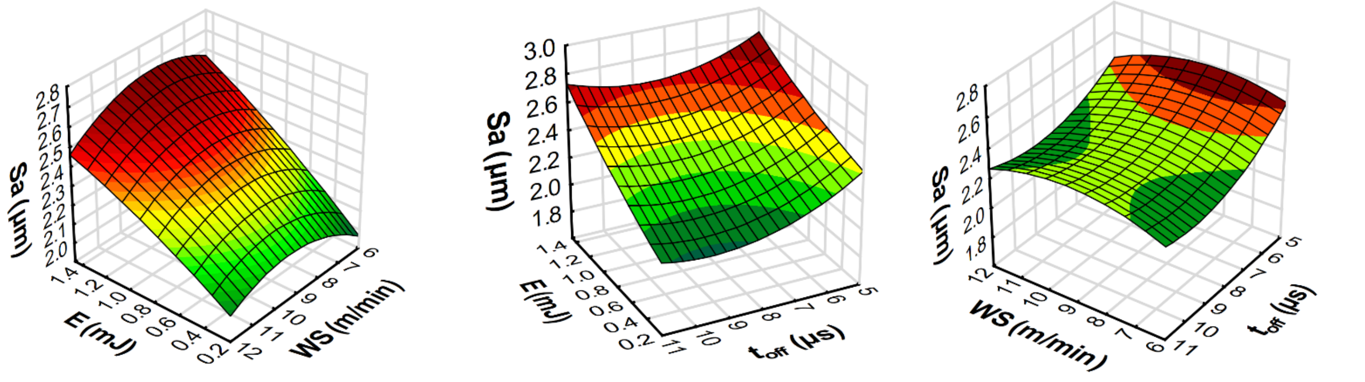

(**a**) (**b**) (**c**)

Figure S2. Estimated response plots for ANN model of roughness *Sa*: (a) constant t_{off} = 8 µs; (b) constant WS = 9 m/min; and (c) constant E = 0.7 mJ

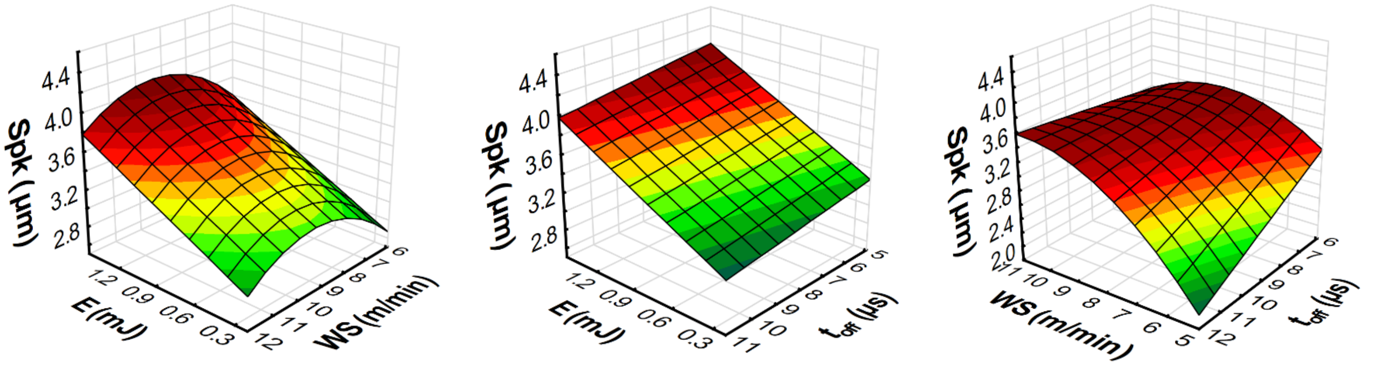

(**a**) (**b**) (**c**)

Figure S3. Estimated response plots for RSM model of roughness *Spk*: (a) constant t_{off} = 8 µs; (b) constant WS = 9 m/min; and (c) constant E = 0.7 mJ

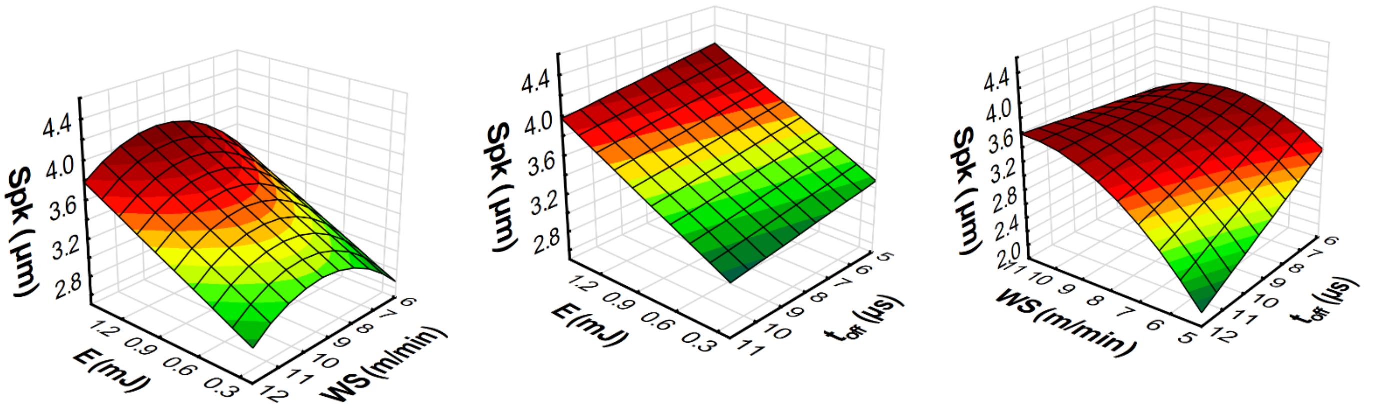

(**a**) (**b**) (**c**)

Figure S4. Estimated response plots for ANN model of roughness *Spk*: (a) constant t_{off} = 8 µs; (b) constant WS = 9 m/min; and (c) constant E = 0.7 mJ

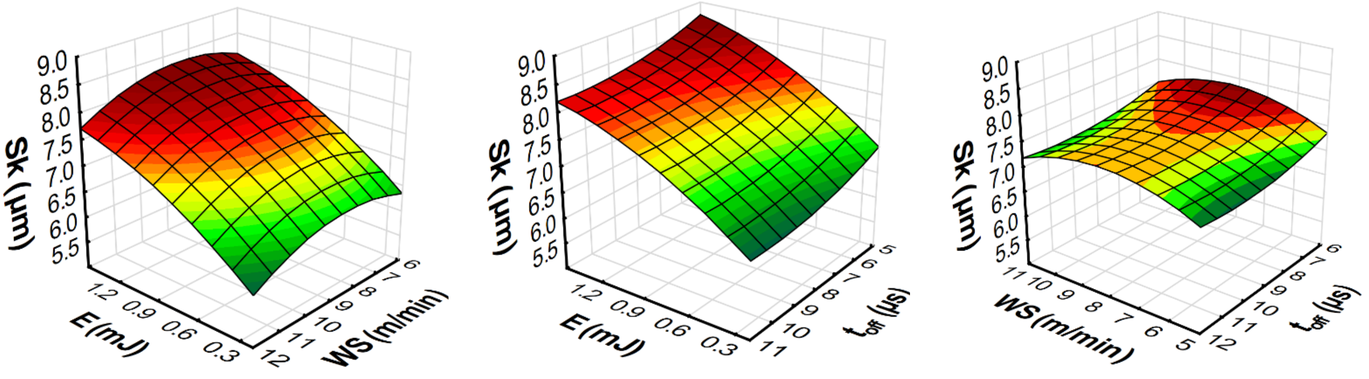

(**a**) (**b**) (**c**)

Figure S5. Estimated response plots for RSM model of roughness *Sk*: (a) constant t_{off} = 8 μs; (b) constant WS = 9 m/min; and (c) constant E = 0.7 mJ

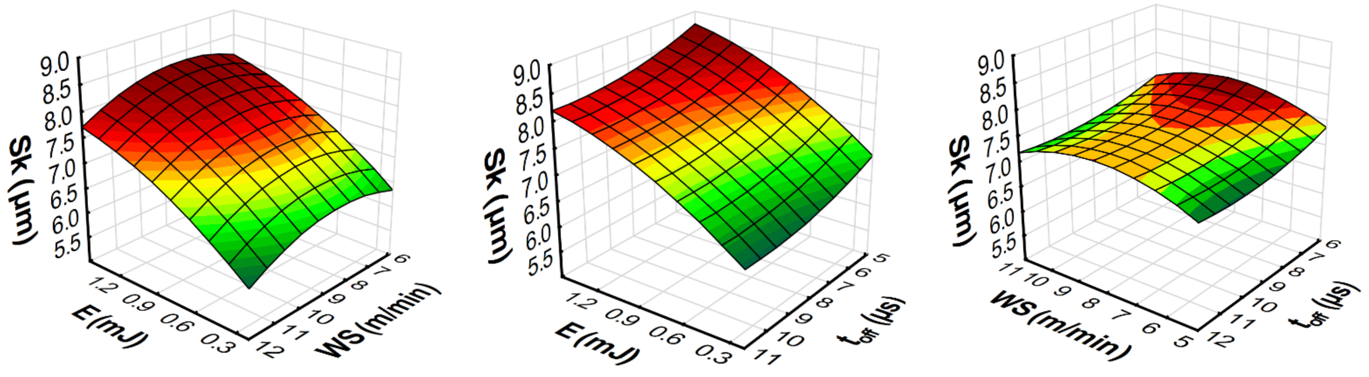

(**a**) (**b**) (**c**)

Figure S6. Estimated response plots for ANN model of roughness *Sk*: (a) constant t_{off} = 8 μs; (b) constant WS = 9 m/min; and (c) constant E = 0.7 mJ

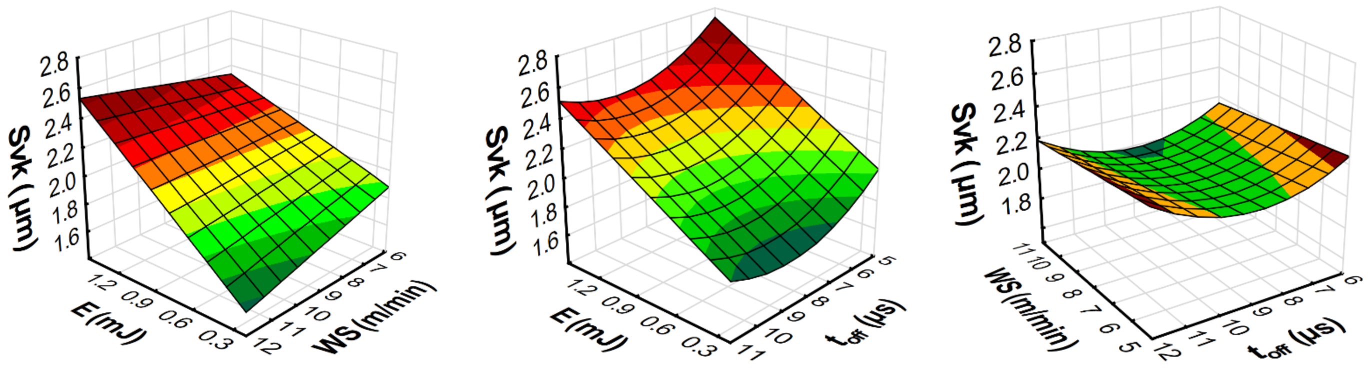

(**a**) (**b**) (**c**)

Figure S7. Estimated response plots for RSM model of roughness *Svk*: (a) constant t_{off} = 8 μs; (b) constant WS = 9 m/min; and (c) constant E = 0.7 mJ

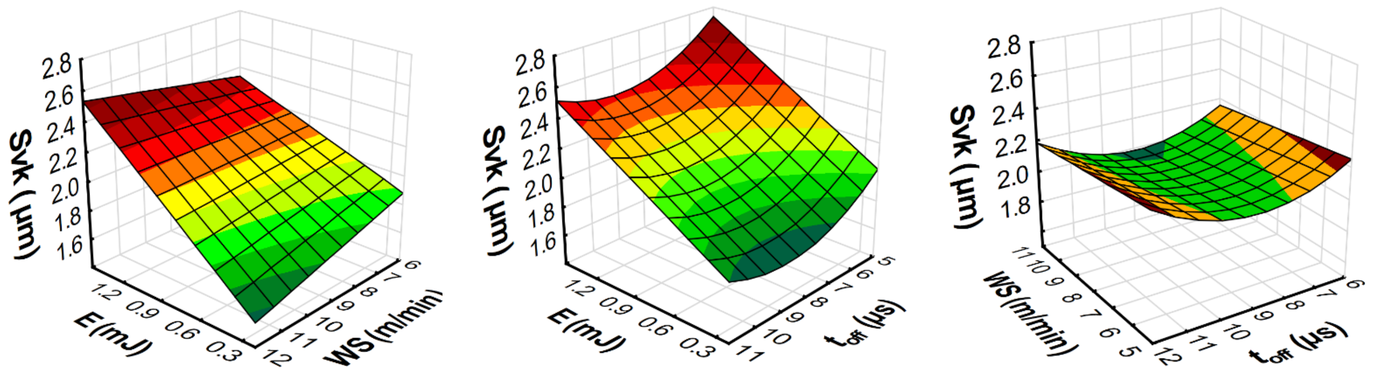

(**a**) (**b**) (**c**)

Figure S8. Estimated response plots for ANN model of roughness *Svk*: (a) constant t_{off} = 8 µs; (b) constant WS = 9 m/min; and (c) constant E = 0.7 mJ

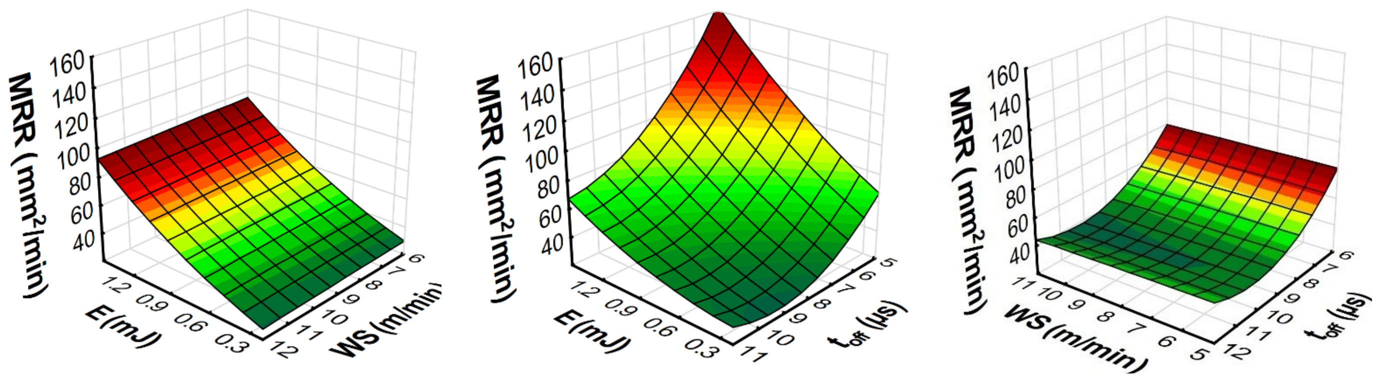

(**a**) (**b**) (**c**)

Figure S9. Estimated response plots for RSM model of *MRR*: (a) constant t_{off} = 8 µs; (b) constant WS = 9 m/min; and (c) constant E = 0.7 mJ

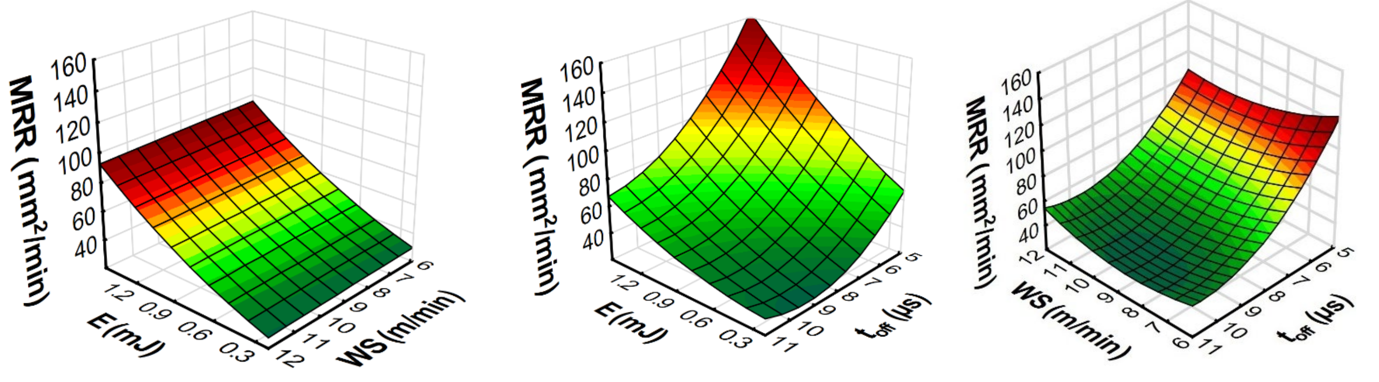

(**a**) (**b**) (**c**)

Figure S10. Estimated response plots for ANN model of *MRR*: (a) constant t_{off} = 8 µs; (b) constant WS = 9 m/min; and (c) constant E = 0.7 mJ

An analysis of the RSM and ANN prediction models of the material removal rate and its graphical interpretation (Figures S1 and S10) indicated that the discharge energy and the time interval made a large contribution to the *MRR* during the micro-WEDM of Inconel 718. *MRR* increased with an increase in the discharge energy and a decrease in the time interval (Figures S10b and S11b). With an increase in discharge energy, the amount of material removed in a single discharge rose, leading to an increase in the *MRR*. These

results coincide with the research of Esteves et al. [56] describing the volume of material removed by a single discharge. The time interval had a direct influence on the frequency of the discharges. An increase in the time interval led to a decrease in the frequency of discharges and ultimately decreased the *MRR*.

This research conducted into micro-WEDM of Inconel 718 has indicated that the wire speed had the least influence on the *MRR* (Figures S10a,c, and S10a,c). Determining the optimal wire speed is important to avoid the effects of wear on the working electrode, which can lead to vibration of the wire and wire breakage.

The results indicate that for the investigated range of wire speeds, the use of speeds of 5 m/min allowed high cutting efficiency to be achieved. A decrease in the wire speed leads to decreased electrode consumption, which has a significant impact on the environment and sustainability.
